# Association of Endoscopic Features of Gastric Mucosa with* Helicobacter pylori* Infection in Chinese Patients

**DOI:** 10.1155/2016/6539639

**Published:** 2016-11-16

**Authors:** Tao Mao, Yan Wang, Fan Yin, Qingxi Zhao, Lin Yang, Xueli Ding, Zibin Tian

**Affiliations:** ^1^Department of Gastroenterology, The Affiliated Hospital of Qingdao University, Qingdao 266003, China; ^2^Qingdao University, Qingdao 266071, China; ^3^Department of Toxicology, Qingdao Municipal Center for Disease Control and Prevention, Qingdao 266033, China

## Abstract

The aim of this study is to identify and consolidate reliable endoscopic features associated with* H. pylori* infection in gastric mucosa, which is one of the major causes of gastric cancer. A total of 256 Chinese patients with symptomatic stomach disturbances were enrolled. Pathological examination was conducted using a light microscope and biopsy specimens stained with hematoxylin-eosin. Endoscopic examination was performed using a high resolution video endoscope. The association between endoscopic features and pathological* H. pylori* diagnosis was compared, and endoscopic features significantly associated with* H. pylori* infection were identified. A total of 14 endoscopic features were observed. Six of the 14 endoscopic features, including mucus on the gastric mucosa, diffuse redness, spotty redness of fundic mucosa, enlarged fold, mucosal edema, and RAC (type D and type I), were highly associated with* H. pylori* infection and were significantly sensitive and specific predictors for* H. pylori* diagnosis. The type R RAC was not significantly associated with* H. pylori* diagnosis. Our results indicate that conventional endoscopy features can be used to diagnose* H. pylori* in Chinese patients and can help determine the risk factor for gastric cancer.

## 1. Introduction

Gastric or stomach cancer typically originates from the mucus-producing cells on the inside lining of the stomach. As early symptoms are rare, it is often diagnosed at an advanced stage. Stomach cancer is more common in certain countries, such as China and Japan, than others, such as the United States [1].

The precise cause of gastric cancers remains unclear, but possible risk factors include smoking, high body mass index, genetic factors, and diets rich in salty or smoked foods [2–4]. Infection with* Helicobacter pylori* (*H. pylori*), a Gram-negative, microaerophilic, and spiral-shaped bacterium, is also known to be a common cause of gastric cancer [5]. The bacterium has evolved various mechanisms to promote its survival in the acidic environment of the stomach and can colonize the gastrointestinal tract [6].

*H. pylori* infection causes chronic inflammation of the gastric mucosa [7] and induces infiltration of mono- and polynuclear cells into the gastric mucosa. Persistent infection can induce atrophic changes and intestinal metaplasia.* H. pylori* infection contributes to a wide variety of upper gastrointestinal tract diseases, including gastroduodenal ulcer, gastric adenocarcinoma, gastric mucosal-associated lymphoid tissue lymphoma, and gastric hyperplastic polyps [8]. Successful eradication of* H. pylori* can improve gastritis and prevent* H. pylori* associated diseases [9]. Eradication of* H. pylori* can also prevent or delay development of precancerous lesions and gastric cancer [10].

*H. pylori* infection can be diagnosed using noninvasive tests such as antibody detection and urea breath test.* H. pylori* specific antibodies can be detected in whole-blood testing inexpensively, but the test has a relatively high false negative rate. The urea breath test uses ^13^C and ^14^C is more sensitive but also more expensive [11, 12].* H. pylori* antigens can be detected in the stool, with similar sensitivity and specificity to antibody testing [13].

Endoscopic atrophy closely correlates with gastric cancer [14, 15]. Many risk factors for gastric cancer, such as* H. pylori* associated gastritis, gastric atrophy, or intestinal metaplasia [5–10], can be diagnosed using endoscopic inspection. If the gastric mucosa appears normal, with none of the aforementioned risk factors, lesions are less likely to be present. In these cases, magnified endoscopy may aid diagnosis.

While endoscopic inspection can identify risk factors for gastric cancer, the endoscopic features of gastric cancer, particularly those of* H. pylori *infection, have not been widely accepted [16–19]. This endeavor is confounded by the complex nature of many endoscopic features, which can be influenced by geographic locations and the ethnicity of patients. For example, features typical of gastric cancer in Asian patients may not be present in Caucasian patients [18]. Endoscopic features may also differ between Japanese and Chinese patients, despite the high incidence of stomach cancer in both populations.

In this study, we analyzed the association between endoscopic features and pathological diagnosis of* H. pylori* infection in Chinese patients. Our goal is to further consolidate the endoscopic diagnosis of* H. pylori*. To the best of our knowledge, this is the first such report concerning the endoscopic diagnosis of* H. pylori* infection in a Chinese population.

## 2. Materials and Methods 

### 2.1. Patient Information

A total of 256 patients, aged between 19 and 83 years, participated in this study, including 118 male and 138 female patients. The patients were admitted to the Affiliated Hospital of Qingdao University (Qingdao, China) with symptomatic stomach disturbances between October and December, 2015, and referred for endoscopic exam. These patients all resided in Shandong province, a peninsula near the Yellow Sea. All patients provided written informed consent prior to biopsy and endoscopic exam. This study was approved by the Ethics Committee of Qingdao University and was carried out in accordance with the Declaration of Helsinki.

Exclusion criteria include (1) history of gastric surgery, (2) gastrectomy, (3) eradication of* H. pylori* infection within one month, (4) treatment with nonsteroidal anti-inflammatory drugs, antiplatelet agents, anticoagulants, steroids, antibiotics, and proton pump inhibitors within 4 weeks prior to entry, (5) severe liver, renal, and cardiopulmonary dysfunctions, and (6) blood diseases including anemia and hemorrhagic tendency.

### 2.2. Pathological Examination

Biopsy specimens were collected from each patient at the following sites: the greater curvature of the antrum; the lesser curvature of the antrum; the lesser curvature of the angulus; the greater curvature of the middle corpus; and the lesser curvature of the middle corpus. Biopsy specimens were stained with hematoxylin-eosin (HE) and examined using a light microscope for the presence or absence of* H. pylori* infection [20]. Each patient was considered* H. pylori* positive if any of the biopsy areas contained* H. pylori*.

### 2.3. Endoscopic Examination

Endoscopic exams were performed using a high resolution video endoscope (GIF-H290, Olympus Co., Japan). More than 50 endoscopic images were recorded at fixed sites in the esophagus, stomach, and duodenum. The following distinctive endoscopic features, based on the Sydney System [20], in images of the antrum, angulus, lesser and greater curvature of the lower body, greater curvature of the upper body, and cardia of the stomach were recorded:

1. Mucus on the gastric mucosa: determined by the visibility of mucus on the surface of gastric mucosa; it can be observed mainly in great curvature of the gastric body; it also can be seen in the nonatrophic mucosa of gastric body
2. Diffuse redness: uniform redness on the entire mucosa of the fundic gland
3. Spotty redness of fundic mucosa: multiple tiny red spots on the fundic gland region; the size of typical spotty redness is usually <1 mm in diameter
4. Patchy redness: localized red areas of various sizes it can be seen in gastric antrum and the atrophic area of gastric body
5. Enlarged fold: fold enlargement, compared to normal fold that is straight, smooth, and approximately 5 mm in diameter
6. Mucosal edema/swelling (fundic/pyloric mucosa):
  1. fundic gland mucosa is soft, thick, and distended
  2. pyloric gland mucosa is soft and convexo-concave
7. Red streak: longitudinal red streaks in the antrum and corpus
8. Regular arrangement of collecting venules (RAC): starfish-like red spots in a regular arrangement, which can be seen in fundic mucosa of the stomach but most obvious in the little curvature of gastric body; we further divide the RAC into three types: (a)* regular type (Type R)*: the diameter and interval of RAC are regular and the 2nd or 3rd levels of branch can be seen; (b)* irregular type (Type I)*: the diameter and interval of RAC are irregular and the 2nd or 3rd levels of branch cannot be seen; (c)* disappear type (Type D)*: RAC is invisible
9. Xanthoma: the yellow-white and well demarcated nodules or plaques in various sizes; xanthoma can be observed in any part of the stomach
10. Fundic gland polyp: sessile polyps of various sizes, usually scattered in the fundic gland region
11. Hyperplastic polyp: polyps in the gastric body and antrum, usually 1 cm in diameter; it is spheroidal, sessile, and occasionally pedunculated; mucosa usually appears normal or erythematous; small erosions may occur
12. Gastric ulcer: ulcers in the stomach
13. Duodenal ulcer: ulcers in the upper area of the small intestine
14. Erosion: (a)* flat type*: flat mucosal defects with whitish-greyish patches; (b)* raised type*: elevated mucosa with white excavation at the center; (c)* hemorrhagic erosion*: erosion with bleeding; (d)* bleeding spot*: punctate, red, or dark ecchymotic spots, streaks, or flecks

Each case was determined by at least 2 well-trained endoscopists. Each patient was considered* H. pylori* positive if at least one of the significant endoscopic features (Table 1) was observed.

### 2.4. Statistical Analysis

Diagnostic accuracy was investigated using MedCalc software (https://www.medcalc.org/). The *χ* ^2^-test was used to evaluate endoscopic and histological parameters. The endoscopic diagnosis of* H. pylori* infection was compared to the histological diagnosis using the following statistical characteristics: specificity, sensitivity, positive predictive value (PPV), negative predictive value (NPV), the area under the curve of receiver operating characteristics (ROC/AUC), and the 95% confidence intervals (CI). *P* < 0.05 was considered to indicate statistical significance.

## 3. Results

*H. pylori* infection was first assessed by pathological diagnosis. Of the 256 Chinese patients included in this study, 44.14% (113/256) were diagnosed with* H. pylori* infection by pathological diagnosis, including 48.31% (57/118) male patients and 40.58% (56/138) female patients. The average age of male patients (51.94 ± 12.51 years) did not differ significantly from the average age of female patients (52.03 ± 11.70 years). Endoscopic diagnosis was highly consistent with the pathological diagnosis. Of the 256 patients, 102 were diagnosed with* H. pylori* by endoscopic examination, 12 of which were* H. pylori* negative according to pathological examination (sensitivity: 90.18%, specificity: 90.91%, PPV: 88.69%, NPV: 92.14%, ROC/AUC: 0.906, *P* < 0.0001). In addition, 11 of the 113 patients found to be* H. pylori* positive by pathological examination were categorized as* H. pylori* negative according to endoscopic examination (false negative rate: 9.73%).

**Figure 1:**
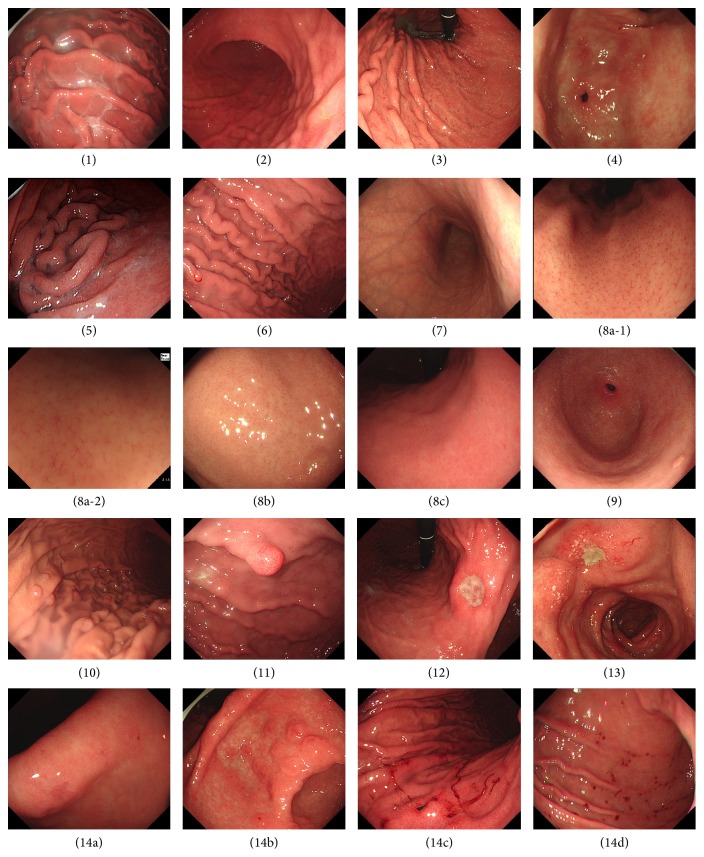
Endoscopic features identified in this study. (1) The mucus on the gastric mucosa, (2) diffuse redness, (3) spotty redness of fundic mucosa, (4) patchy redness, (5) enlarged fold, (6) mucosal edema/swelling (fundic/pyloric mucosa), (7) red streak, (8) regular arrangement of collecting venules (RAC): (8a-1) RAC (type R) observed with conventional endoscope, (8a-2) RAC (type R) observed with magnifying endoscope, (8b) RAC (type I), and (8c) RAC (type D), (9) Xanthoma, (10) fundic gland polyp, (11) hyperplastic polyp, (12) gastric ulcer, (13) duodenal ulcer, and (14) erosion: (14a)* flat type*, (14b)* raised type,* (14c)* hemorrhagic erosion, and* (14d)* bleeding spot*.

Fourteen of the endoscopic features defined, based on the Sydney System [6, 7, 21], were observed in our study, including (1) mucus on the gastric mucosa; (2) diffuse redness; (3) spotty redness of fundic mucosa; (4) patchy redness; (5) enlarged fold; (6) mucosal edema/swelling (fundic/pyloric mucosa); (7) regular arrangement of collecting venules (RAC); (8) red streak; (9) xanthoma; (10) fundic gland polyp; (11) hyperplastic polyp; (12) gastric ulcer; (13) duodenal ulcer; and (14) erosion (Figure 1).

In this patient population, the most reliable endoscopic features of* H. pylori* diagnosis were spotty redness, enlarged fold, RAC, and mucosal edema (marked with an “*∗*” in Table 2). They were all highly specific and sensitive indicators of* H. pylori* infection. The specificity/sensitivity of each feature were spotty redness: 95.8%/61.06%, enlarged fold: 92.23%/60.15%, RAC: 90.21%/86.73%, and mucosal edema: 89.51%/72.27% (Table 2).

We further categorized RAC into three distinct types, types R, I, and D (see Section 2.3). The rates of infection in patients in which these features were observed were type R: 10.49% (15/143); type I: 85.71% (24/28); and type D: 87.06% (74/85). The rate of infection in patients with type D and type I RAC were similar (*χ*2 ID = 0.07, *P* > 0.05) and were both significantly higher than the rate of infection in patients with type R RAC (*χ*2 RI = 112.7, *P* < 0.01; *χ*2 RD = 147.46, *P* < 0.01).

Diffuse redness and mucus on the gastric mucosa were also highly associated with* H. pylori* infection, with 95% specificity and >50% sensitivity. All four types of erosion (flat, raised, hemorrhagic, and bleeding spot) were highly specific (>92%) but insensitive predictors of infection, possibly due to the small number of erosion negative patients in this sample. Gastric ulcer, duodenal ulcer, hyperplastic polyp, and fundic gland polyp were also highly specific predictors of infection, with high PPVs. However, few* H. pylori *positive patients presented with these features, only 12 with gastric ulcer, 19 with duodenal ulcer, 2 with hyperplastic polyp, and 5 with fundic gland polyp. It is therefore difficult to evaluate the significance of these endoscopic features for* H. pylori* infection diagnosis.

Only five cases of red streak and four cases of patchy redness were observed, and none of these patients were* H. pylori *positive. Thirteen cases of xanthoma were identified, of which six patients were* H. pylori* positive. However, 12 of these 13 patients had previously been diagnosed with* H. pylori* infection, and thus xanthoma likely resulted from previous* H. pylori* infection, rather than current infection.

## 4. Discussion

Endoscopic features have been reported to be useful tools for the diagnosis of* H. pylori *infection of gastric mucosa. However, the predictive capacity of these features may vary between ethnicities. In this study, we identified 14 gastric mucosa endoscopic features in 256 Chinese patients with strong symptomatic stomach disturbances. Consistent with a previous study [22], we found the endoscopic features spotty redness and enlarged fold to be highly associated with* H. pylori *infection. In addition, we found mucus on the gastric mucosa, diffuse redness, mucosal edema, and RAC to also be associated with* H. pylori *infection, suggesting that these features may be unique indicators of* H. pylori *infection in Chinese patients. Of the three RAC types, we found that a much higher rate of* H. pylori *infection was associated with type I and type D. This finding is consistent with a previous study in which magnifying endoscopy was used in a Chinese population [23], suggesting that the type I and type D RAC could be used as reliable endoscopic features for* H. pylori *diagnosis. It is also important to note that the type R RAC was not found to be associated with* H. pylori *infection, a finding that may reduce application of additional and unnecessary medical examinations.

It has been suggested that erosion is an indicator of absence of* H. pylori* infection [21]. In our study, about 55% of patients in which erosion was detected were* H. pylori* negative (Table 2), indicating that erosion is not an accurate indicator of the presence or absence of* H. pylori* infection. Patch redness has also been suggested as an indicator of* H. pylori* eradication [24]. In this study we observed patchy redness in only 4 patients, and although all 4 patients were* H. pylori* negative, we feel that this number of cases is insufficient to draw a reliable conclusion.

*H. pylori* infection has been previously associated with increased incidence of both gastric ulcer and duodenal ulcer. Nonsteroidal anti-inflammatory drugs were reported to reduce the incidence of duodenal ulcer but not gastric ulcer [25–27]. In this study, gastric ulcers were only observed in 12 patients, and duodenal ulcers were only observed in 19 patients, so although our results are consistent with previous findings, again, this number of cases is insufficient to draw a reliable conclusion. This limitation also applies to several other endoscopic features, including patchy redness, hyperplastic polyp, red streak, xanthoma, fundic gland polyp, and hyperplastic polyp. In the future, studies enrolling a larger sample size, from multiple hospital facilities, may provide more informative results.

In summary, we identified 14 endoscopic features in 256 Chinese patients with symptomatic stomach disturbances. Our results suggest that at least 6 of the 14 endoscopic features, including mucus on the gastric mucosa, diffuse redness, spotty redness of fundic mucosa, enlarged fold, mucosal edema, and RAC (type D and type I), are highly associated with* H. pylori* infection and could be used as reliable indicators for* H. pylori* diagnosis. In addition, we found that type R RAC is likely not associated with* H. pylori *infection. This finding may help to reduce unnecessary medical examinations. Additional studies with larger sample sizes and more diverse populations may help to characterize the relationship between endoscopic features such as diffuse redness and erosion and* H. pylori* diagnosis. We believe that our results contribute to the development of rapid* H. pylori* infection diagnosis, one of the risk factors for gastric cancer.

## Figures and Tables

**Table 1:** Significant endoscopic features used for *H. pylori* diagnosis in relation to pathological examination.

Endoscopic features	Endoscopy exam	Pathology exam
(1) Mucus on the gastric mucosa	+	Hp positive
	−	Hp negative
(2) Diffuse redness	+	Hp positive
	−	Hp negative
(3) Spotty redness of fundic mucosa	+	Hp positive
	−	Hp negative
(4) Patchy redness	+	Hp positive
	−	Hp negative
(5) Enlarged Fold	+	Hp positive
	−	Hp negative
(6) Mucosal edema	+	Hp positive
	−	Hp negative
(7) Red streak	+	Hp positive
	−	Hp negative
(8) RAC		
*Type R*	+	Hp negative
*Type I*	+	Hp positive
*Type D*	+	Hp positive
(9) Xanthoma	+	Hp positive
	−	Hp negative
(10) Fundic gland polyp	+	Hp positive
	−	Hp negative
(11) Hyperplastic polyp	+	Hp positive
	−	Hp negative
(12) Gastric ulcer	+	Hp positive
	−	Hp negative
(13) Duodenal ulcer	+	Hp positive
	−	Hp negative
(14) Erosion		
*Flat type*	−	Hp positive
	+	Hp negative
*Raised type*	−	Hp positive
	+	Hp negative
*Hemorrhagic erosion*	−	Hp positive
	+	Hp negative
*Bleeding spot*	−	Hp positive
	+	Hp negative

Note: the “+” or the “−” sign in the column of “Endoscopy exam” indicates the presence (+) or absence (−) of the endoscopic feature, which corresponds to the pathology exam of *H. pylori* positive (Hp positive) or *H. pylori* negative (Hp negative).

**Table 2:** Associations between endoscopic and histological evaluations for *H. pylori* infection.

		Hp^+^	Hp^−^	Total	Sensitivity (%)	Specificity (%)	PPV (%)	NPV (%)	ROC/AUC	*P*
					(95% CI)	(95% CI)	(95% CI)	(95% CI)	(95% CI)	
^*∗*^Mucus on the gastric mucosa	+	58	7	65	53.33	95.1	89.23	71.2	0.732	<0.001
	−	55	136	191	41.7–60.8	90.2–98.0	79.1–95.6	64.2–77.5	0.673–0.785	
^*∗*^Diffuse redness	+	65	6	71	57.52	95.8	91.54	74.65	0.767	<0.001
	−	48	137	185	47.9–66.8	91.1–98.4	82.5–96.8	67.1–80.2	0.710–0.817	
^*∗*^Spotty redness of fundic mucosa	+	69	6	75	61.06	95.8	92.99	75.69	0.784	<0.001
	−	44	137	181	53.4–70.1	91.1–98.4	83.4–97.0	68.8–81.7	0.729–0.833	
Patchy redness	+	0	4	4	28	100	100	56.56	0.514	<0.05
	−	113	139	252	0.8–7.0	96.8–100	31.2–100	50.2–62.8	0.451–0.577	
^*∗*^Enlarged fold	+	68	12	80	60.18	92.25	85.99	74.57	0.762	<0.001
	−	45	131	176	56.5–69.3	86.6–96.1	76.4–92.8	67.5–80.8	0.765–0.813	
^*∗*^Mucosal edema	+	72	15	97	72.27	89.51	84.48	80.33	0.81	<0.001
	−	31	128	159	63.4–80.5	83.3–94.0	75.7–91.0	73.3–86.2	73.3–86.2	
Red streak	+	0	5	5	100	2.8	44.84	100	0.514	<0.05
	−	113	138	251	96.5–100	0.8–7.0	38.6–51.2	39.8–100	0.451–0.577	
^*∗*^RAC	+	98	15	113	86.73	90.21	87.5	89.6	0.815	<0.001
	−	128	15	143	79.1–92.4	84.1–94.5	79.9–93.0	83.4	0.839–0.921	
Xanthoma	+	6	7	13	5.31	95.1	46.33	55.97	0.052	>0.05
	−	136	107	243	2.0–11.2	90.2–98.0	19.2–74.8	49.5–62.3	0.439–0.565	
Fundic gland polyp	+	5	21	26	14.69	95.58	72.42	58.54	0.551	<0.05
	−	108	122	230	9.3–21.6	90.0–98.5	50.0–88.8	52.0–65.0	0.488–0.613	
Hyperplastic polyp	+	2	0	2	1.77	100	100	56.3	0.509	>0.05
	−	111	143	254	0.2–6.2	97.5–100	15.8–100	15.8–100	45.96–62.491	
Gastric ulcer	+	9	3	12	7.96	97.9	74.97	57.38	0.529	<0.05
	−	104	140	244	3.7–14.6	94.0–99.6	42.8–94.5	50.9–63.7	0.466–0.592	
Duodenal ulcer	+	13	6	19	13.5	95.8	68.39	57.8	0.537	<0.05
	−	100	137	237	6.3–18.9	91.1–98.4	43.4–87.4	51.2–64.1	0.473–0.599	
Erosion										
*Flat type*	+	105	131	236	8.39	92.92	48.36	56.21	0.507	>0.05
	−	8	12	20	4.4–14.2	86.5–96.9	25.6–71.6	49.6–62.6	0.444–0.569	
*Raised type*	+	112	139	251	2.8	99.12	71.54	56.34	0.51	>0.05
	−	1	4	5	0.8–7.0	95.2–100	19.4–98.6	50.0–62.6	0.477–0.572	
*Hemorrhagic erosion*	+	111	142	253	13.89	93.81	66.28	57.4	0.528	>0.05
	−	2	1	3	96.2–100	0.2–6.2	38.2–50.8	16.9–99.8	0.442–0.568	
*Bleeding spot*	+	106	126	232	12.89	93.81	66.28	57.4	0.528	>0.05
	−	7	17	24	7.1–18.4	87.7–97.5	37.6–80.1	50.8–63.8	0.465–0.591	

The “+” and “−” signs indicate the presence (+) or absence (−) of an endoscopic feature; “Hp^+^” indicates *H. pylori *positive, and “Hp^−^” indicates *H. pylori *negative. NPV: negative predictive value; PPV: positive predictive value; ROC/AUC: area under the curve of receiver operating characteristics. 95% CI: 95% confidence intervals.
